# A Compact Dual-Band Millimeter Wave Antenna for Smartwatch and IoT Applications with Link Budget Estimation

**DOI:** 10.3390/s24010103

**Published:** 2023-12-24

**Authors:** Parveez Shariff Bhadrvathi Ghouse, Pallavi R. Mane, Sangeetha Thankappan Sumangala, Vasanth Kumar Puttur, Sameena Pathan, Vikash Kumar Jhunjhunwala, Tanweer Ali

**Affiliations:** 1 Department of Electronics and Communication Engineering, Manipal Institute of Technology, Manipal Academy of Higher Education, Manipal 576104, India; parveez.bg@learner.manipal.edu (P.S.B.G.); palvi.mane@manipal.edu (P.R.M.); vasanth.puttur@manipal.edu (V.K.P.); 2 Department of Information and Communication Technology, Manipal Institute of Technology, Manipal Academy of Higher Education, Manipal 576104, India; sangeetha.ts@manipal.edu (S.T.S.); sameena.bp@manipal.edu (S.P.); 3 Department of Electrical and Electronics Engineering, Manipal Institute of Technology, Manipal Academy of Higher Education, Manipal 576104, India; vikas.kumar@manipal.edu

**Keywords:** dual-band, IoT, link margin, millimeter wave antenna, SAR, smartwatch

## Abstract

Advancement in smartwatch sensors and connectivity features demands low latency communication with a wide bandwidth. ISM bands below 6 GHz are reaching a threshold. The millimeter-wave (mmWave) spectrum is the solution for future smartwatch applications. Therefore, a compact dual-band antenna operating at 25.5 and 38 GHz is presented here. The characteristics mode theory (CMT) aids the antenna design process by exciting Mode 1 and 2 as well as Mode 1–3 at their respective bands. In addition, the antenna structure generates two traverse modes, TM_10_ and TM_02_, at the lower and higher frequency bands. The antenna measured a bandwidth (BW) of 1.5 (25–26.5 GHz) and 2.5 GHz (37–39.5 GHz) with a maximum gain of 7.4 and 7.3 dBi, respectively. The antenna performance within the watch case (stainless steel) showed a stable |S_11_| with a gain improvement of 9.9 and 10.9 dBi and a specific absorption rate (SAR) of 0.063 and 0.0206 W/kg, respectively, at the lower and higher bands. The link budget analysis for various rotation angles of the watch indicated that, for a link margin of 20 dB, the antenna can transmit/receive 1 Gbps of data. However, significant fading was noticed at certain angles due to the shadowing effect caused by the watch case itself. Nonetheless, the antenna has a workable bandwidth, a high gain, and a low SAR, making it suitable for smartwatch and IoT applications.

## 1. Introduction

Smartwatches (SWs) have become an integral part of our lives, as they are used for health and fitness parameter tracking, entertainment, phone calls, controlling other Internet of Things (IoT) devices, Global Positioning System (GPS) navigation, and so on [1]. In 2023 alone, it is estimated that there are 210.8 million SW users globally, which is expected to grow to 229.51 million users by 2027 [2]. With the rapid development in technology, SWs are loaded with sophisticated sensors to record physiological data such as respiratory [3], cardiac activity [4,5], and blood pressure [6]. The telemetry of these data from SW to the remote diagnostic center via the internet requires seamless connectivity. A precisely tailored antenna with a compact design, a low specific absorption rate (SAR), a good radiation pattern, a high gain, and an ease of embedding in SWs can bridge this gap.

The 0.472λ_0_ × 0.472λ_0_ (with λ_0_ at 2.44 GHz) antenna can house a ground plane with four vertical metal rims [7]. This results in a low SAR of 1.24 and 1.44 W/kg for SW applications at 2.44 and 3.5 GHz, respectively. In [7], a metal ring-shaped antenna of 23 mm for bezel SW is proposed. The antenna efficiency and low electric-field distribution on the wrist were improved by placing a metal plate at the bottom. However, the actual bandwidth (BW) was 66 and 101 MHz at 1.55 and 2.4 GHz, respectively. As described in [8], multiple inverted L-shaped structures were etched on a 0.3λ_0_ × 0.35λ_0_ substrate operating at 0.88–0.96, 2.4–2.48, 3.3–50, and 5.1–5.8 GHz, achieving SAR in the range of 0.84–1.21 W/kg. In the later designs, the SW screen effect was not considered. Generally, the SW screen has a printed circuit board (PCB) with perfect electric conductor (PEC) cladding that supports the SW screen. Thus, embedding the antenna below the screen affects the radiation pattern. In [9], these effects were studied by considering flexible printed circuit (FPC) communication strips. The FPC strips carried signals to/from the PCB board to the SW screen. Two conductive tape-based structures were carved on a flexible printed circuit (FPC) to provide shielding. The length of these tapes was tuned to achieve resonances at 1.6, 2.4, 3.5, and 4.8 GHz with a structure dimension of 0.23λ_0_ × 0.28λ_0_.

The above-discussed structures have a large antenna profile with narrow bandwidths. The forthcoming SW is expected to support video calling and video streaming. Therefore, the millimeter wave spectrum that offers a wide bandwidth with low latency is the solution for personal area IoT applications [10]. In [11], a triple-band CPW monopole antenna was proposed for IoT applications with bandwidths of 22–30, 37–39, and 56.5–61 GHz. They achieved a SAR in the 0.9–0.62 W/kg range and a gain of 5.29, 7.49, and 9 dBi. Another design in [12] proposed a triple-band antenna for IoT applications. The structure with a dimension of 2.8λ_0_ × 2.35λ_0_ (with λ_0_ at 28 GHz) combined two varied-length monopoles with a conducting stub at the bottom. The achieved bandwidths were 445 MHz, 657 MHz, and 5.14 GHz at 3.5, 5.8, and 28 GHz, and an antenna gain of 1.86, 2.55, and 4.41 dBi.

Very few designs exist that are suitable for SW IoT applications. Therefore, we designed a compact dual-band 0.51λ_0_ × 0.51λ_0_ (with λ_0_ at 25.5 GHz) antenna with measured antenna bandwidths of 1.5 (25–26.5 GHz) and 2.5 GHz (37–39.5 GHz) and gains of 7.4 and 7.9 dBi, respectively. The antenna evolved through characteristic mode analysis (CMA), which was first proposed by Garbacz [13,14], providing a physical insight into an arbitrarily shaped antenna. Recently, CMA has been used to aid the design and configuration of MIMO, circular polarization, dual-polarization [15], and reconfigurable antennas [16,17]. The electromagnetic behavior of the antenna is mathematically modeled using weighted eigenvalue (λ_n_), which depicts the number of significant modes that contribute to the resonance and provides information on the orthogonal electric field and surface current distribution of these modes [18]. It also provides information about the modes impedance characteristics, be they purely resistive, inductive, or capacitive. This information aids in tuning the antenna close to the ideal radiating conditions. The solution to λ_n_ conceptualizes the radiator’s resonance, inductive, or capacitive mode [19], which can be altered by modifying the antenna structure.

The antenna performance was studied for SW applications by placing it inside a watch case with the material properties of stainless steel. The top of the watch case was covered with a reinforced glass of PTFE material. The proposed antenna with stainless steel at the bottom resulted in the lowest SAR of 0.063 and 0.0206 W/kg at respective bands. The SW and mobile phone link budget analysis was performed at 0.5 m for different watch angles. This showed satisfactory results with data supports of up to 1 Gbps. Therefore, the major contributions of this study are as follows:

1. the design and development of a compact dual-band antenna with the aid of characteristic mode theory to comprehend the resonant modes and their resultant radiation pattern;
2. an analysis of the performance of the proposed antenna for smartwatch applications by embedding it inside the watch case;
3. a study of the RF energy exposure to the human body caused by the proposed antenna with a watch case;
4. an estimation of the data handling capacity of the proposed antenna for a smartwatch at various angles in three-dimensional space.

## 2. Characteristic Mode-Based Antenna Methodology

The characteristic mode analysis (CMA) is a rapid development tool applied to the antenna development stages to comprehend its performance and radiation pattern. The structure was modified to achieve the desired results. CMA estimates the effective modes of an arbitrarily shaped antenna structure, where the first five modes are mostly found to be significant. However, modifying the structure and changing the feed position can generate undesired modes. In Stage 1 of the antenna design, an initial rectangular patch antenna was designed based on the design equations in [20] for a resonating frequency of 25.5 GHz.

**Figure 1:**
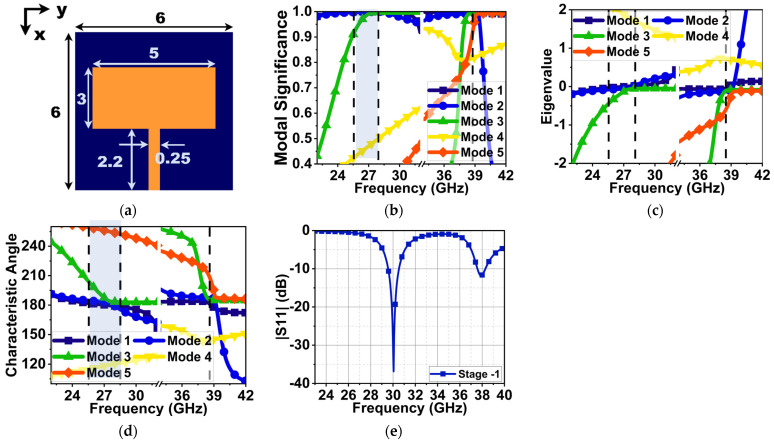
CMA of Mode 1–5 and reflection coefficients of the Stage 1 MS antenna. (**a**) Stage 1 antenna (dimensions in mm), (**b**) modal significance, (**c**) eigenvalues, (**d**) characteristic angle, and (**e**) reflection coefficient with port excitation.

From the above calculations, the approximate patch width and length were 4.65 and 3.718 mm, respectively. However, to retain compactness and to study the modified structure behavior, the patch width and length were chosen to be 5 and 3 mm in the first stage, as shown in Figure 1a. For CMA, a 0.51λ_0_ × 0.51λ_0_ lossless Roger 5880 substrate with a thickness of 0.25 mm and relative permittivity $\left(\varepsilon_{r}\right)$ of 2.2 was selected. The electromagnetic simulation software CST v21 was used to analyze antenna development stages without port excitation. The antenna was designed to generate dual-band resonances at 25.5 and 38 GHz. Therefore, the CMA was performed separately for each of these frequencies. During CMA, the total surface current on the antenna structure of the patch and ground plane was estimated by considering the sum of the eigenvector $\left(J_{n}\right)$ using Equation (1) [15,18].

(1) $$J=\sum_{n}\frac{V_{n}J_{n}}{1+j\lambda_{n}}\;\;\;\;\;\;\;\;\;$$

where $V_{n}$ is the model-excitation coefficient. The $V_{n}$ along with model significance (MS) defines the significant modes at desired frequencies, where $MS=\left|\frac{1}{1+j\lambda_{n}}\right|$. In Figure 1b, the modal significance graph indicates that Mode 1 and 2 are significant at 25.5 GHz, Mode 1–3 are significant at 28 GHz with a wide bandwidth, and Mode 1–5 are significant at 38.5 GHz with a narrow bandwidth. The convergence of these modes eigenvalue (λ_n_) to zero indicates that the modes are resonant. However, the modes with an eigenvalue (λ_n_) > or <0 are non-resonant inductive and capacitive modes. These modes store the energy in the form of electric and magnetic fields in the near-field region. The eigenvalue (λ_n_) indicates that Mode 1–3 and 1–4 at 28 and 38.5 GHz are resonances, as shown in Figure 1c.

The characteristics angle (CA) is another feature of CMA, which represents the resonant and non-resonant modes by analyzing the phase lag between the electric field and surface current on the antenna structure. It is represented in terms of angle $\left(\alpha_{n}\right)$. The characteristics angle in Figure 1d depicts that Mode 1–3 from 27–28.5 GHz have zero eigenvalues, resulting in $\alpha_{n}=180^{{}^{\circ}}$, which means these modes are resonant modes with good impedance matching. Beyond this frequency, the Mode 1 and 2 surface current’s phase lag from the electric field led to an inductive (non-resonant mode) mode. At 38.5 GHz, a poor impedance matching with a narrow bandwidth can be seen due to the summation of phase-lead and phase-lag of Mode 3 and 5 and Mode 2, respectively. The port excitation corroborates in the same manner, which indicates that, at 30 GHz, a good impedance matching |S11| of 37 dB with a bandwidth of 29.5–30.5 GHz is achieved, as shown in Figure 1e. However, at 38 GHz, due to inductive and capacitive Mode 2, 3, and 5, a poor impedance matching of 12 dB can be seen. Therefore, the CMA estimation for the first stage closely agrees with the port excitation results.

**Figure 2:**
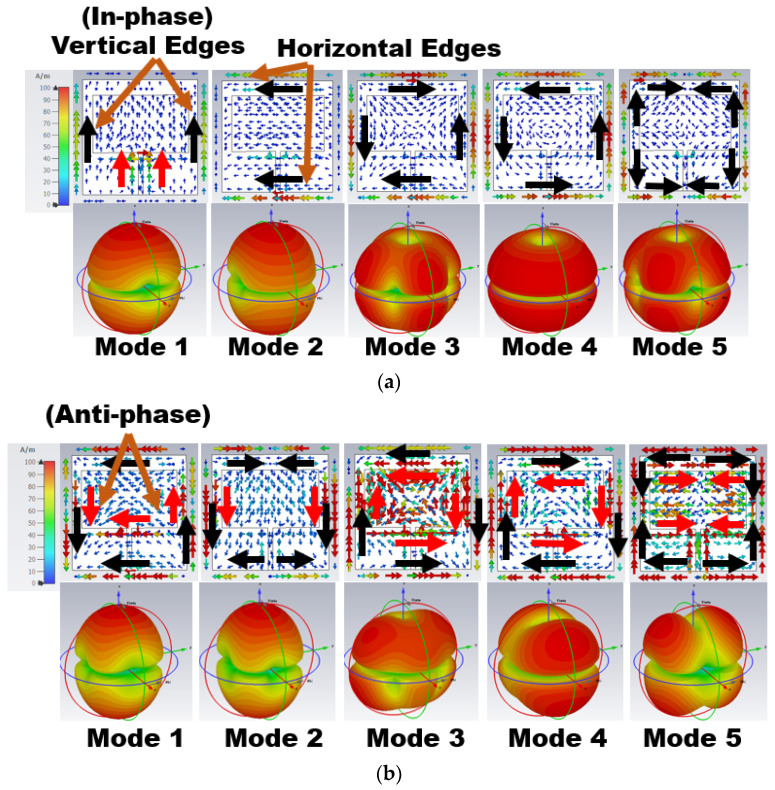
Eigenvector current *J_n_* on the surface of Stage 1 and its respective radiation pattern at (**a**) 25.50 GHz and (**b**) 38 GHz.

The sum of all modes of eigenvector currents *(J_n_)* represents the total current on the antenna structure. In Figure 2, the eigenvector current *(J_n_)* is concentrated over the antenna surface edges. Based on the *J_n_* movement and direction, the respective modes characterize the radiation behavior. The eigenvector current and radiation pattern of the respective modes at 25.50 and 38 GHz are shown in Figure 2a,b, respectively. In Figure 2a, Mode 1 and 2 have in-phase vector currents (currents on the parallel edges are in the same direction) along the vertical/horizontal edges of the ground (black arrow) and radiator (red arrow). Mode 1 has an in-phase current along the vertical edges, causing a bidirectional radiation pattern with a null in the azimuthal plane at ϕ = 0°.

Similarly, Mode 2 has an in-phase current at horizontal edges, providing a bidirectional radiation pattern with nulls in the azimuthal plane at ϕ = 90°. Mode 3–5 have an anti-phase eigenvector current (current in the opposite direction of the parallel edges) along the antenna edges. Thus, a quad/end-fire radiation pattern is formed, as seen in Figure 2a.

Similar effects can be observed in Figure 2b at 38 GHz but with the most current in the radiator. Mode 1 and 2 have an in-phase current on one set of parallel edges and an anti-phase current on the other, resulting in bidirectional quad radiation. However, Mode 3–5 have an anti-phase current, resulting in end-fire radiation.

**Figure 3:**
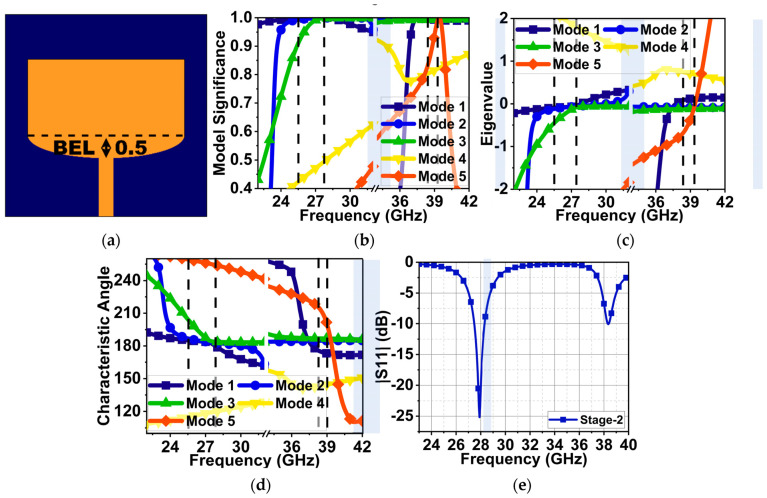
CMA of Mode 2–5 and reflection coefficients of the Stage-1 MS antenna. (**a**) Stage-1 antenna (dimensions in mm), (**b**) modal significance, (**c**) eigenvalues, (**d**) characteristic angle, and (**e**) reflection coefficient with port excitation.

As discussed above, the first resonance occurred at 30 GHz with good impedance matching and the second at 38 GHz with poor impedance matching. Further, in Stage 2, to decrease the first resonance to the expected frequency of 25.5 GHz and improve the impedance matching at 38 GHz, a semi-elliptical stub was added at the bottom of the radiator, as displayed in Figure 3a. From the CMA of Stage 2, the results of the MS in Figure 3b indicate that Mode 1–3 drifted from 30 to 28 GHz due to structure modification. However, at 25.5 GHz, only Mode 1 and 2 were found to be significant. On the other hand, Mode 1, 2, 3, and 5 drifted slightly higher, from 38.5 to 39 GHz. The eigenvalue of these modes converged to zero, as shown in Figure 3c, at respective frequencies, resulting in resonant modes with bandwidths of 24.1–26, 27–28, and 37.5–39 GHz. The characteristic angle shows a 180° phase lag between the surface current and the electric field at 25.5, 28, and 39 GHz of the resonant modes. However, the Mode 5 surface current led the electric field by 210° until 39 GHz, abruptly changing to a phase lag of 120° after 39 GHz. The Mode 4 surface current had a phase lag of 150° from 36 to 40 GHz. Due to these phase differences in Mode 4 and 5, the structure could not radiate complete energy at 39 GHz. As a result, an impedance mismatch occurred. The port excitation substantiated the CMA. The results indicate the occurrence of resonances at 28 and 38 GHz with impedance matching of 25 and 10.5 dB, as shown in Figure 3e.

**Figure 4:**
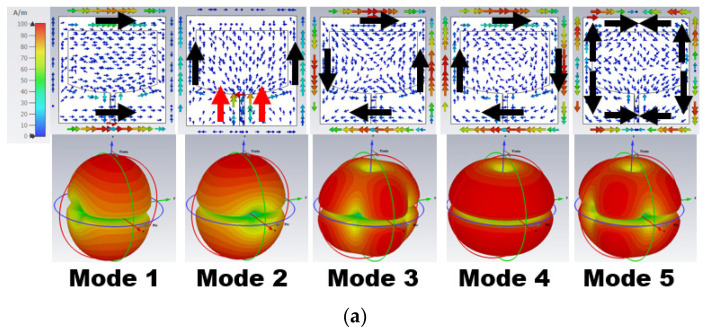

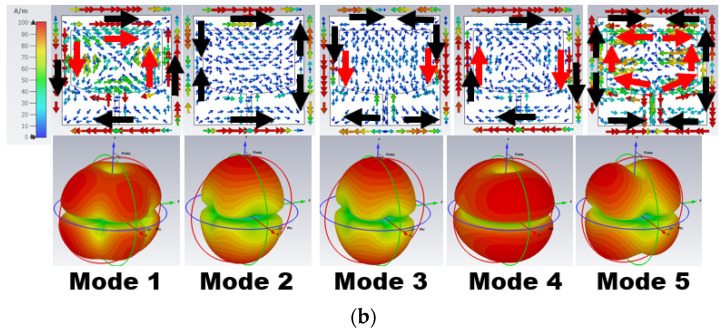
Eigenvector current *J_n_* on the surface of Stage 2 and its respective radiation pattern at (**a**) 25.50 GHz and (**b**) 38 GHz.

Figure 4 represents the eigenvector current at 25.5 and 38 GHz. From the above eigenvalue and characteristic angle, only Mode 1 and 2 are resonant, but these are insufficient to generate the resonance at 25.5 GHz. This is evident from the eigenvector current in Figure 4a, as most of the current is in the ground plane rather than the radiator. At 38 GHz, Mode 1–3 have an in-phase current generating resonance, as shown in Figure 4b. However, an anti-phase current arose due to the non-resonant Mode 4 and 5, causing impedance matching.

**Figure 5:**
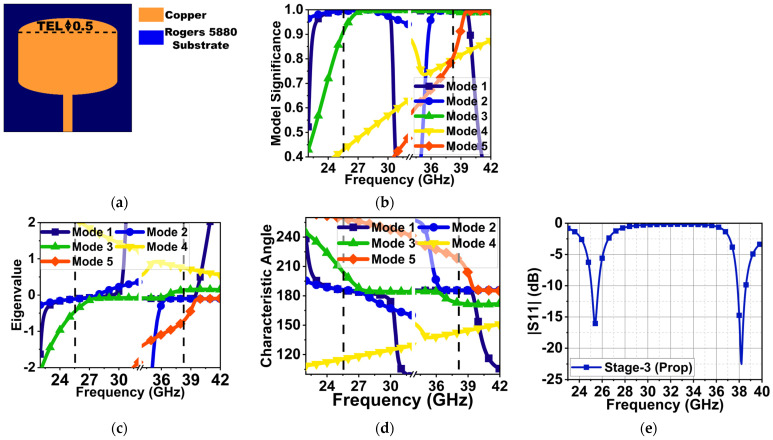
CMA of Mode 2–5 and reflection coefficients of the Stage-3 MS antenna. (**a**) Stage-1 antenna (dimensions in mm), (**b**) modal significance, (**c**) eigenvalues, (**d**) characteristic angle, and (**e**) reflection coefficient with port excitation.

Stage 3 with a semi-elliptical stub at the top of the radiator was introduced, as displayed in Figure 5a, to lower the resonance from 28 to 25.5 GHz and to improve the impedance matching at 38 GHz. This improved the significance of Mode 1–3 at 25.5 and 38 GHz, as shown in Figure 5b. The convergence of these modes to zero in the eigenvalue, as shown in Figure 5c, represents resonant modes (Mode 3 at 25.5 GHz is partially resonant) with bandwidths of 25–27 and 37–39 GHz. Mode 1–3 have an approximately 180° phase lag between the surface current and the electric field, as represented by the characteristics angle in Figure 5d. The modification to the structure significantly suppressed Mode 4 and 5 at 25.5 and 38 GHz. With the port excitation, dual-resonance can be seen in Figure 5e at 25.5 and 38 GHz with good impedance matching of 17.5 dB and 22.5 dB. It achieved a 25–25.9 GHz and 37.9–38.9 GHz bandwidth.

**Figure 6:**
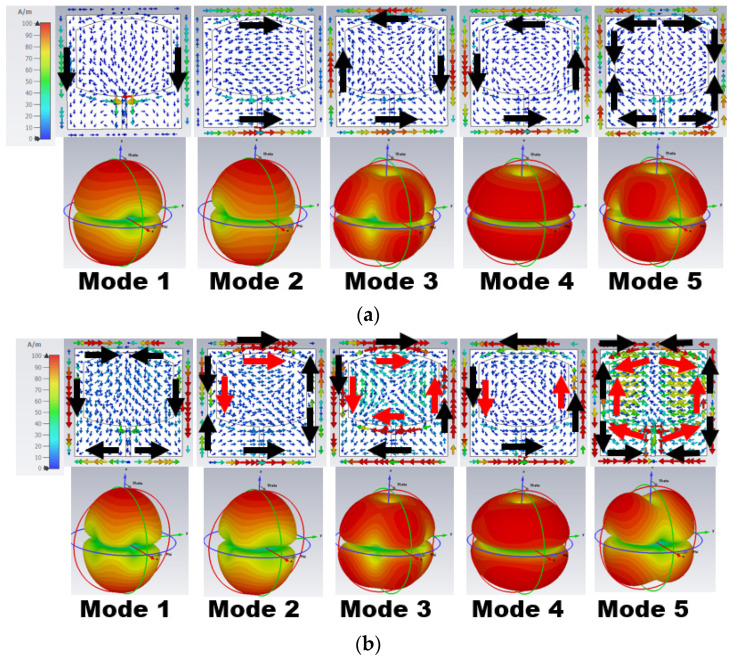
Eigenvector current *J_n_* on the surface of Stage 3 and its respective radiation pattern at (**a**) 25.50 GHz and (**b**) 38 GHz.

The eigenvector current of Mode 1 and 2 at 25.5 GHz has an in-phase current resulting in bidirectional radiation, as shown in Figure 6a. At 38 GHz (Figure 6b), Mode 1 has an in-phase current along the vertical edges and an anti-phase current along the horizontal edges, resulting in bidirectional quad radiation. Similarly, in Mode 2, there are in-phase currents along horizontal edges and anti-phase currents along vertical edges. Though Mode 5 is largely capacitive, its surface current impacts the radiation patterns.

## 3. Current Distribution with Port Excitation

**Figure 7:**
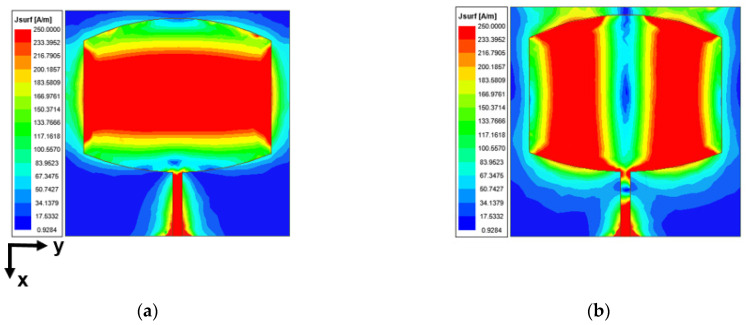
Current distribution with port excitation at (**a**) 25.5 GHz and (**b**) 38 GHz.

From the above CMA and port excitation, it is shown that the structure generates dual resonance at 25.5 and 38 GHz. Figure 7 indicates the current oscillation at the respective frequencies when the port is excited. At 25.5 GHz, the current oscillation is along the ±*x*-axis (i.e., vertical oscillation) because the patch length at the center is close to half-wavelength at 25.5 GHz, which is 0.4λ_0_ in this case. The current is at its maximum in the feed and the center of the patch along the *x*-axis. This gives rise to the TM_10_ mode at this frequency. However, at 38 GHz, the current oscillation in Figure 7b can be seen along the ±*y*-axis because the patch width is close to half-wavelength at 38 GHz, which is 0.6λ_0_ in this case. Two current maximums along the patch width give rise to the TM_02_ mode at this frequency.

## 4. Parametric Analysis of Antenna Elliptical Structure

**Figure 8:**
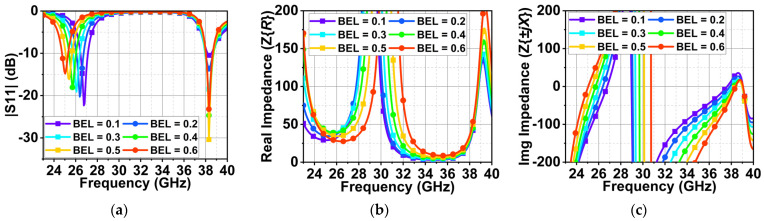
Variation in (**a**) reflection coefficient |S11| due to a change in the variable BEL, (**b**) Real and (**c**) imaginary impedance values at respective BEL values.

In this section, parametric analysis of two variables, BEL and TEL, is performed to study the effect of resonance change due to the radiator length change and its impact on the antenna impedance. Here, one variable is maintained at 0.5 mm, and the other varies from 0.1 to 0.6 mm. In the first case, TEL is kept at 0.5 mm, and BEL is varied. With the BEL at 0.1 mm, the overall length of the patch is reduced. Thus, the first resonance occurs at 27 GHz with an impedance matching of 22 dB because the impedance is purely real with 50 Ω at 27 GHz, as shown in Figure 8b. However, the reduced length impacts the impedance at 38 GHz with a poor impedance matching of 11 dB, as shown in Figure 8a. This is because the impedance at this frequency has a small inductance effect with a total impedance Z of 42 + j37 Ω. Further, with the patch length increase and a BEL from 0.2 to 0.6 mm, the first resonance drifts to lower frequencies with little decrease in impedance matching. This is because, as the patch length increases, the resonance decreases. At the second resonance, there is no effect on frequency drift. However, impedance matching is improved to a minimum of 30 dB.

**Figure 9:**
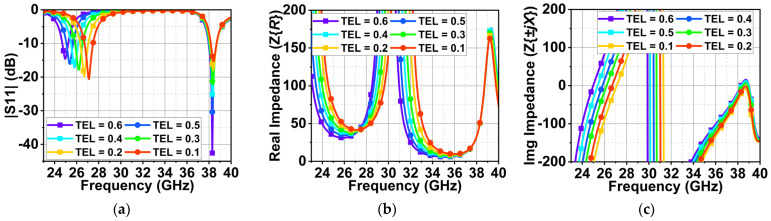
Effect of varying TEL on (**a**) the reflection coefficient |S_11_, (**b**) the real part of the impedance, and (**c**) the imaginary parts of impedance.

In the second case, BEL is maintained at 0.5 mm, and TEL varies from 0.1 to 0.6 mm. A similar response can be observed compared to that of BEL. However, for TEL = 0.1 mm, the impedance matching at 25.5 and 38 GHz is good with 20 and 15 dB, as shown in Figure 9a. At 25.5 and 38 GHz, the impedance is real and close to 50 Ω, as displayed in Figure 9b,c. With a further increase in TEL from 0.2 to 0.6 mm, the first resonance is decreasing, and the second resonance impedance is improving, similar to the earlier case.

## 5. Results and Discussion

### 5.1. Reflection Coefficients and Radiation Patterns in Free Space

**Figure 10:**
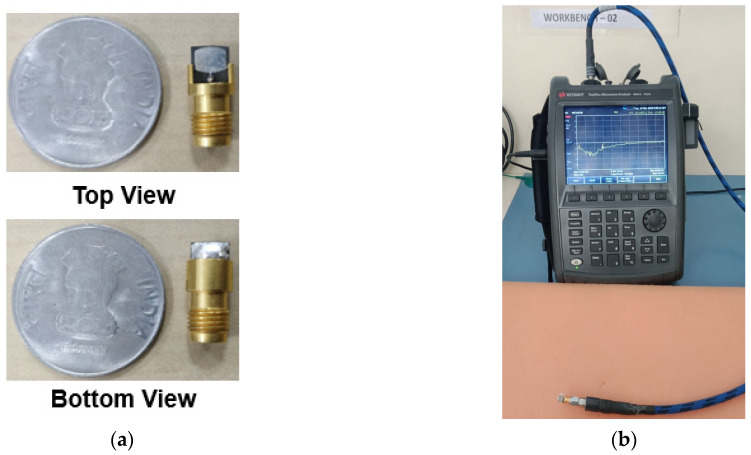
Photos of the antenna and the reflection coefficient measurements. (**a**) Photos of the antenna and (**b**) the measurement setup of the |S11| parameter.

**Figure 11:**
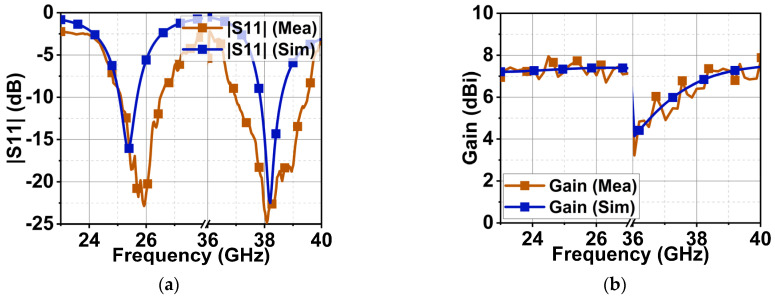
Simulated and measured reflection coefficients and gain. (**a**) |S_11_|and (**b**) gain.

**Figure 12:**
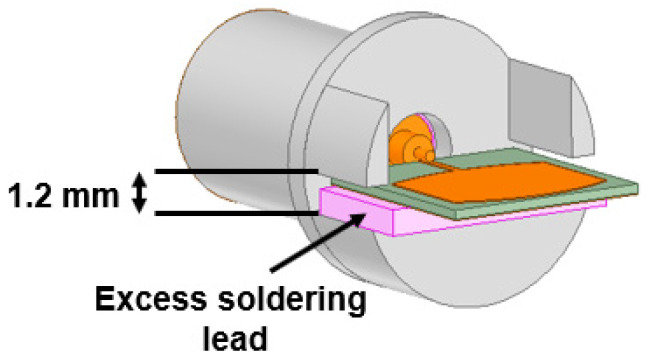
3D representation of the proposed antenna with the SMA connector.

The proposed antenna was fabricated, as shown in Figure 10a. The antenna reflection coefficient |S_11_| was measured using the Keysight vector network analyzer, which ranges from 300 KHz to 44 GHz. The connectors used also supported RF signal feeding up to 50 GHz. The |S_11_| measurement setup is shown in Figure 10b. The CMA results indicate that Mode 1 and 2 are resonant, with Mode 3 partially contributing to the resonance at 25.5 GHz with a bandwidth of 24–27 GHz. At 38 GHz, Mode 1–3 are resonant with a bandwidth of 26.2–39.3 GHz. The high-frequency simulation software (HFSS) version 21.0 results illustrate the occurrence of two resonances at 25.5 and 38 GHz with a bandwidth of 750 MHz (25–25.75 GHz) and 1 GHz (37.92–38.92 GHz). The measured |S11| shows resonance at 25.85 and 38 GHz with bandwidths of 1.5 GHz (25–26.5 GHz) and 2.5 GHz (37–39.5 GHz), as displayed in Figure 11a. The deviation in the measured results may be due to fabrication tolerance, cable, and connector losses. The procured connectors (the best available in the market) also have a 1.2 mm gap between the center signal and ground lead. When the antenna is fitted inside, a gap of 0.94 mm still exists that is filled with excessive soldering lead, as shown in Figure 12. Above all, the proposed antenna is very small and lacks industrial standard soldering tools, necessitating manual soldering that causes a lead spread on the ground plane. All of this impacts the antenna’s performance, causing deviations in the measurement results, as shown in Figure 11. However, the measured results are considerable compared to the simulated results. The antenna has an almost constant simulated and measured gain of 7.4 and 7.9 dBi at first resonance. Nonetheless, at second resonance, the gain almost linearly increases with the maximum simulated and measured gains of 7.4 and 7.3 dBi, as shown in Figure 11b.

**Figure 13:**
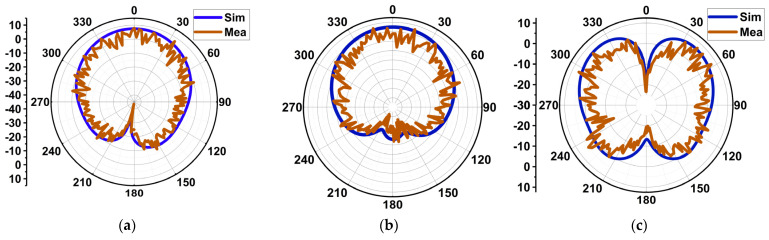
Simulated and measured radiation patterns. (**a**) The XZ-plane at 25.5 GHz, (**b**) the YZ-plane at 25.5 GHz, and (**c**) the YZ-plane at 38 GHz.

As discussed in the current distribution section, the proposed antenna generates TM_10_ and TM_02_ at 25.5 and 38 GHz, respectively. Due to these modes, the radiation is a single beam in the broadside direction at 25.5 GHz, whereas two beams at 38 GHz are tilted at 36°. Therefore, the radiation pattern is measured on the XZ- and YZ-planes at 25.5 GHz (Figure 13a,b), whereas, at 38 GHz, only the YZ-plane is considered (Figure 13c). The simulated and measured half-power-beamwidths (HPBWs) at 25.5 GHz are 78° and 62° on the XZ-plane. On the YZ-plane, they are 82° and 62°. At 38 GHz, the simulated and measured HPBWs are 58° and 45°.

### 5.2. Antenna Performance with Watch Case

**Figure 14:**
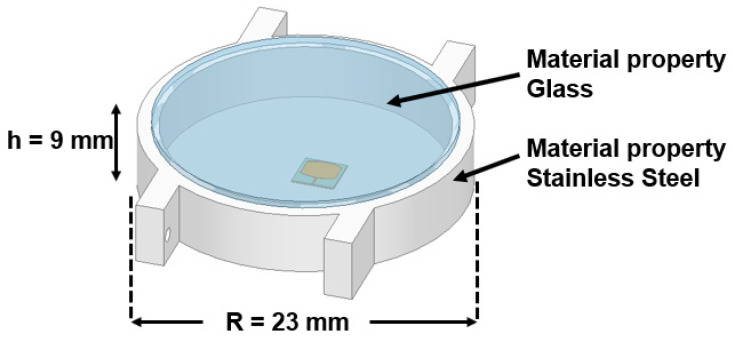
Model of the smartwatch with the antenna inside.

**Figure 15:**
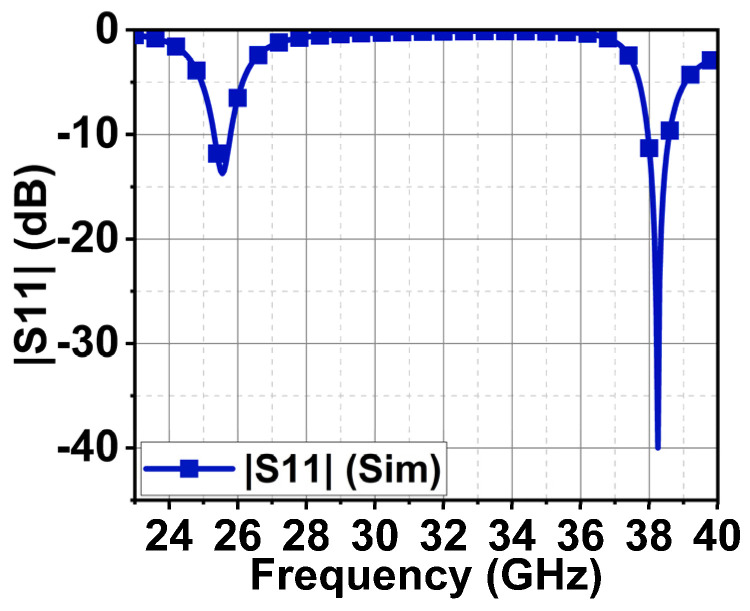
Reflection coefficient |S_11_| antenna response when placed inside the watch case.

**Figure 16:**
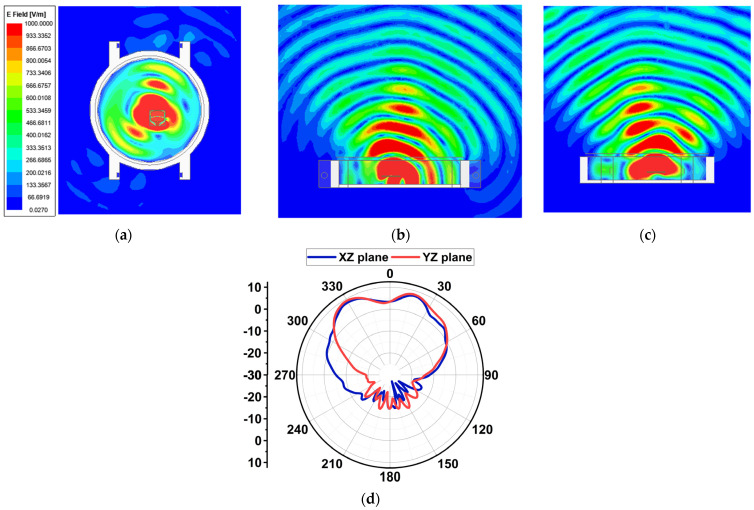
Electric field distribution and radiation patterns at 25.5 GHz with the antenna inside the watch case. (**a**) The XY-plane, (**b**) the XZ-plane, (**c**) the YZ-plane, and (**d**) the radiation pattern on the YZ-plane.

**Figure 17:**
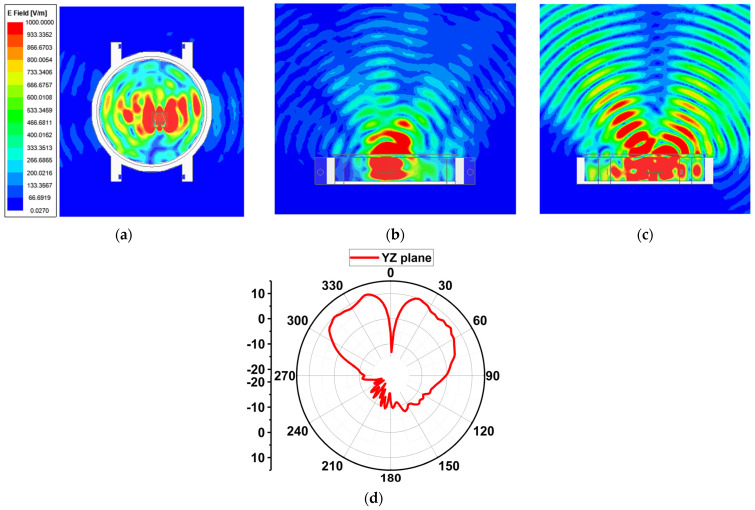
Electric field distribution and radiation patterns at 38 GHz with the antenna inside the watch case. (**a**) The XY-plane, (**b**) the XZ-plane, (**c**) the YZ-plane, and (**d**) the radiation pattern on the YZ-plane.

Since the proposed antenna is for SW applications, its performance was studied by placing it inside a round dial watch case of 23 mm radius and a height of 9 mm, as displayed in Figure 14. The watch body case was considered as stainless steel with a relative permittivity of 1. The watch top was covered with reinforced glass of PTFE material with a relative permittivity of 2.5. The antenna was placed at a height of 2.5 mm from the bottom of the watch case. At 25.5 GHz, a slight decrease in impedance occurs due to the formation of a negative image current on the metallic surface of the watch case [21,22]. However, at 38 GHz, the impedance is significantly increased. The reflection coefficient |S_11_| is shown in Figure 15. Nonetheless, other than an increase in the front-to-back ratio of radiation due to the metallic casing, the antenna performance is not strongly affected. The electric field distribution in and around the watch case is presented in Figure 16 and Figure 17. On the XY-plane at 25.5 GHz, the E-field is concentrated inside the watch case mostly along the *x*-axis due to TM_10_, as illustrated in Figure 16a. Outside the case, a minor E-field distribution can be seen at the front and back of the watch lug. Figure 16b,c show the E-field along the XZ- and YZ-planes. It is shown that the E-field intensity is in the broadside direction. The radiation pattern in Figure 16d shows a directional beam with an HPBW of 30° on the XZ- and YZ-planes with a maximum gain of 9.9 dBi. In the case of 38 GHz, the E-field distribution is on the *y*-axis of the XY-plane due to TM_02_, as depicted in Figure 17a. The E-field is mostly concentrated inside the case, and some intensity can be seen along and opposite the watch crown side. Figure 17b,c show the E-field on the XZ- and YZ-planes. The intensity of the field is maximum on the YZ-plane in two directions, approximately ±36°. The radiation pattern with two beams on the YZ-plane is shown in Figure 17d with an HPBW of 46° and a maximum gain of 10.9 dBi.

### 5.3. SAR Analysis

**Figure 18:**
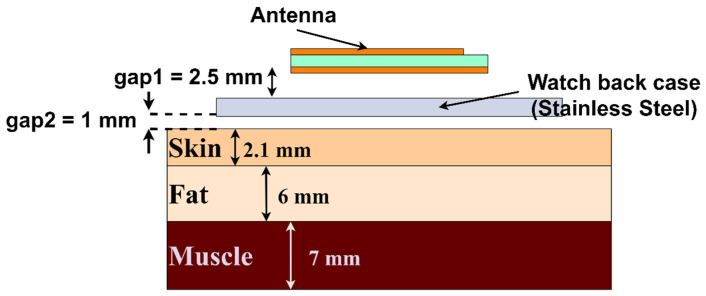
Phantom model of human tissue to analyze the RF energy penetration.

**Figure 19:**
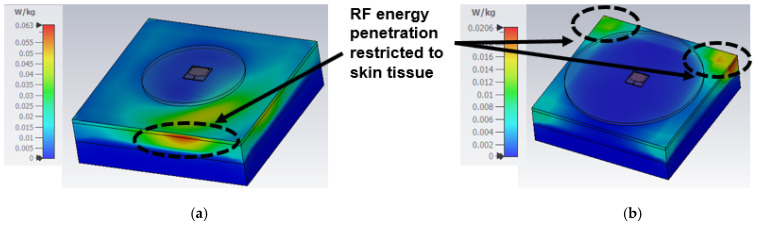
SAR analysis results (**a**) at 25.5 GHz and (**b**) at 38 GHz.

This section presents the study of the RF energy absorption on human tissue generated from the designed antenna, as the proposed design is for wearable smartwatch applications. As per new guidelines of the International Commission for Non-Ionizing Radiation Protection (ICNIRP), the absorption rate is mainly at the skin layer with less than 1 cm for frequencies >6 GHz [23,24]. However, a three-layer phantom model was developed with skin, fat, and muscle to study the absorption rate, as displayed in Figure 18. The dielectric properties of human tissue vary over the body and the different frequencies. Therefore, the tissue parameters are considered from a verified dataset of the IT’IS foundation [25]. Table 1 presents the dielectric property, electrical conductivity, and density of tissues considered for our analysis at 25.5 and 38 GHz. Due to the limitation of computation resources, the watch case is replaced by the watch back case only, which is of a 1 mm thickness and a 23 mm radius. The antenna is placed 2.5 mm above this case. The analysis shows that the SAR is 0.063 and 0.0206 W/kg at 25.5 and 38 GHz, respectively, as displayed in Figure 19. There are two reasons for the low SAR value: (i) The antenna has a full ground plane, and (ii) the antenna is backed by a watch back case that behaves as a reflector for electromagnetic energy. Consequently, the results are satisfactory, suggesting that the antenna is suitable for wearable smartwatch applications.

### 5.4. Link Budget Analysis

**Figure 20:**
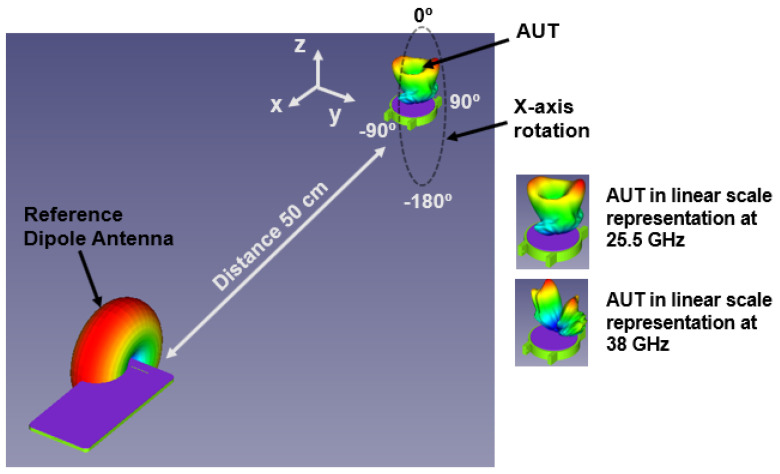
Link budget estimation environment under line-of-sight conditions.

The smartwatch on the human wrist experiences complex three-dimensional movement during various day-to-day activities. Therefore, error-free transmission and reception are essential for seamless, smooth communication between smartwatches and mobile/IoT devices during these movements. A link budget was studied between the mobile and proposed smartwatch antenna under a line-of-sight (LOS) environment at various rotational degrees. A standard dipole antenna was considered as a reference antenna (transmitting antenna) inside the mobile case with a gain ($G_{td}$) of 1 dBi. The receiving antenna is the proposed antenna (antenna under test (AUT)). The receiver antenna gain ($G_{tr}$) is 9.9 and 10.9 dBi at 25.5 and 38 GHz (placed inside the watch case, as discussed in the previous section). In our case, the reference antenna and AUT were separated by a 50 cm distance. The AUT was stationary but rotated over the *x*-axis from 0° to ±180°, as depicted in Figure 20. The received signal at various rotational angles was computed using the shooting and bouncing ray technique in HFSS Savant, considering the losses such as the path loss exponent effect and the shadowing effect (LS) [26]. Therefore, Equation (2) [26,27] can calculate the received power as

(2) $$P_{rw}=P_{td}+G_{td}+G_{tr}-LS\;\;\;\;$$

In the analysis, the power of the transmitting antenna ($P_{td}$) considered is 30 mW. The required power ($R_{Pw}$) to estimate the link budget for an ideal binary-phase-shift-keying (BPSK) with an energy-to-noise ratio ($E_{b}/N_{0}$) of 9.6 dB is given by Equation (3):

(3) $$R_{Pw}\;\left(dB\right)=\frac{E_{b}}{N_{0}}\;\left(dB\right)+KT+B_{r}\;\left(dB\right)\;\;$$

where *K* is the Boltzmann constant with a value of $1.38\times 10^{-23}$, *T* is the temperature in Kelvin (in our case, 290 K), and $B_{r}$ represents the various bit rates supported for reliable communication. The link margin is the difference between the actual received power $P_{rw}$ and required power $R_{Pw}$ given by Equation (4) [27,28]:

(4) $$LM\;\left(dB\right)=P_{rw}\;\left(dB\right)-R_{Pw}\;\left(dB\right)\;\;\;\;\;\;\;\;\;\;\;\;\;$$

**Figure 21:**
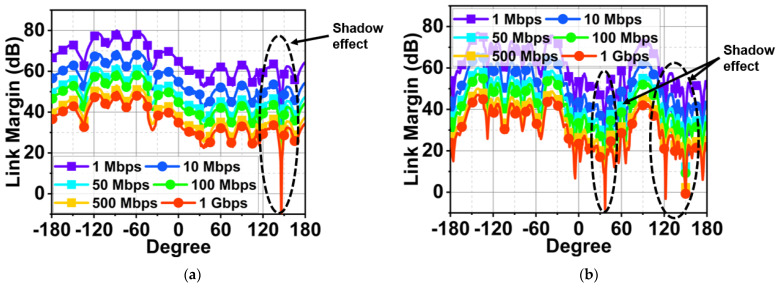
Estimated link budget between the ideal dipole antenna and the proposed antenna at 50 cm distance for various rotational angles for the two bands. (**a**) 25.5 GHz and (**b**) 38 GHz.

Assuming that a link margin of 20 dB is sufficient for error-free communication, the proposed antenna with the highest data rate of 1 Gbps is supported with a link margin of >20 from 0° to −180° angle orientation, as depicted in Figure 21a,b. At these angles, both the reference and AUT antenna have good LOS conditions; in fact, direct LOS occurs at −90°. From 0° to 180°, specifically from 45° to 135°, the AUT antenna experiences a shadowing effect due to the metallic case of the watch. As a result, the link margin significantly deteriorates, as shown in Figure 21a,b. Nonetheless, the performance of the proposed antenna is satisfactory, with a data rate support of 1 Gbps.

### 5.5. Comparative Analysis

The proposed antenna performance was compared with the existing designs in Table 2. The proposed antenna is relatively compact compared to most of the other antennas in the table. The antenna also has a comparatively optimal gain. The proposed antenna with the bottom watch case has the lowest SAR value. Most of the antennas in the table are planar antennas; however, due to partial ground/defected ground structure (to improve the bandwidth), the antenna structure replicates monopole behavior. As a result, these are labeled as monopole antennas. In our case, the antenna has a full ground plane depicting a perfect patch antenna. The proposed antenna’s link budget was also analyzed, and it was demonstrated that the antenna can deliver 1 Gbps of data at a 20 dB link margin, which other designs have not performed.

## 6. Future Scope

In line with the presented research work, other contributions can be made: (1) The RF energy exposure by the proposed antenna with SWs can be studied in real time by mimicking the phantom model. (2) In the current work, the effect of the SW screen was neglected. In future work, the effect of the SW screen, which is supported by PCB and has PEC properties, can be considered in the analysis. (3) Further, link margin analysis can be performed by considering the human body between SWs and mobile phones, since at the mmWave level, the human body creates obstruction, leading to NLOS communication.

## 7. Conclusions

This article presents a compact dual-band antenna operating at 25.5 and 38 GHz bands. Due to its compact design, it is suitable for smartwatch and IoT applications. The antenna structure has generated two modes at 25.5 GHz and three modes at 38 GHz, Mode 1 and 2 and Mode 1–3, which are analyzed using characteristic mode theory. Apart from these modes, the antenna generated traverse TM_10_ and TM_02_ modes at the respective bands. The antenna has a good measured bandwidth of 1.5 and 2.5 GHz with gains of 7.4 and 7.9 dBi. Due to traverse modes, the antenna has radiation in the broadside with single and dual beams at their respective bands. The antenna showed a sustainable |S_11_| level when tested with the watch case. This also improved the gain to 9.9 and 10.9 dBi. The results of SAR analysis indicate minimal RF energy penetration to human tissue, which makes it suitable for wearable applications. The link budget estimation demonstrated the highest data rate transfer of 1 Gbps for a link margin of 20 dB.

## Figures and Tables

**Table 1:** Human tissue properties at different frequencies.

		25.5 GHz		38 GHz	
Tissue	Density (kg/m^3^)	Permittivity	Electrical Conductivity (s/m)	Permittivity	Electrical Conductivity (s/m)
Skin	1109	18	24	12.3	31
Fat	911	6.34	4.66	5.33	6.36
Muscle	1090	26.2	31.1	19.1	41.8

**Table 2:** Comparative analysis of proposed antenna with existing designs.

Ref	Ant Type	Dim (mm^2^)	Res (GHz)	BW (GHz)	Gain (dBi)	SA (W/kg)	Radiation	LM Analysis
[11]	Mono	0.37λ_0_ × 27λ_0_	27/38/60	22–30/38–39.5/56–61	5.29/7.49/9	0.9/0.62/0.74	Bi-Dir	No
[29]	Mono	1.1λ_0_ × 0.86λ_0_	28.5/38	26–40.5	3.8	NA	Bi-Dir	No
[30]	Mono	2.41λ_0_ × 2λ_0_	60	58.2–60.5	9.9	NA	Multi-Beam	No
[31]	Mono	1.36λ_0_ × 1.22λ_0_	28/38	22–29/37–40	5	NA	Omni	No
[32]	Mono	0.75λ_0_ × 1.24λ_0_	37	36.7–37.4	NA	NA	Broadside	No
[33]	Mono	1.12λ_0_ × 1.12λ_0_	28/40	24–32/38–41	4.5	NA	Bi-Dir	No
Proposed	Patch	0.51λ_0_ × 0.51λ_0_	25.5/38	25–26.5/37–39.5	7.4/7.9	0.063/0.0206	Broadside	Yes

Note: Ref: reference; Ant: antenna; Dim: dimension; Res: resonance; LM: link margin; BW: bandwidth.

## Data Availability

All the data are contained within the manuscript.

## References

[1] Chandel R.S., Sharma S., Kaur S., Singh S., Kumar R. (2022). Smart Watches: A Review of Evolution in Bio-Medical Sector. Mater. Today Proc..

[2] Global: Smartwatches Number of Users 2018–2027. https://www.statista.com/forecasts/1314339/worldwide-users-of-smartwatches.

[3] Hao T., Bi C., Xing G., Chan R., Tu L. (2017). MindfulWatch: A Smartwatch-Based System for Real-Time Respiration Monitoring During Meditation. Proc. ACM Interact. Mob. Wearable Ubiquitous Technol..

[4] Xintarakou A., Sousonis V., Asvestas D., Vardas P.E., Tzeis S. (2022). Remote Cardiac Rhythm Monitoring in the Era of Smart Wearables: Present Assets and Future Perspectives. Front. Cardiovasc. Med..

[5] Kwon J., Jo Y.-Y., Lee S.Y., Kang S., Lim S.-Y., Lee M.S., Kim K.-H. (2022). Artificial Intelligence-Enhanced Smartwatch ECG for Heart Failure-Reduced Ejection Fraction Detection by Generating 12-Lead ECG. Diagnostics.

[6] He J., Ou J., He A., Shu L., Liu T., Qu R., Xu X., Chen Z., Yan Y. (2022). A New Approach for Daily Life Blood-Pressure Estimation Using Smart Watch. Biomed. Signal Process. Control.

[7] Xu Z., Wang Y. (2023). Design of Dual-Band Antenna for Metal-Bezel Smartwatches with Circular Polarization in GPS Band and Low Wrist Effect. IEEE Trans. Antennas Propagat..

[8] Liao C.-T., Yang Z.-K., Chen H.-M. (2021). Multiple Integrated Antennas for Wearable Fifth-Generation Communication and Internet of Things Applications. IEEE Access.

[9] Xiao B., Wong H., Wu D., Yeung K.L. (2021). Design of Small Multiband Full-Screen Smartwatch Antenna for IoT Applications. IEEE Internet Things J..

[10] Mallat N.K., Ishtiaq M., Ur Rehman A., Iqbal A. (2022). Millimeter-Wave in the Face of 5G Communication Potential Applications. IETE J. Res..

[11] Ahmad S., Boubakar H., Naseer S., Alim M.E., Sheikh Y.A., Ghaffar A., Al-Gburi A.J.A., Parchin N.O. (2022). Design of a Tri-Band Wearable Antenna for Millimeter-Wave 5G Applications. Sensors.

[12] Ruchi, Patnaik A., Kartikeyan M.V. (2022). Compact Dual and Triple Band Antennas for 5G-IOT Applications. Int. J. Microw. Wirel. Technol..

[13] Garbacz R., Turpin R. (1971). A Generalized Expansion for Radiated and Scattered Fields. IEEE Trans. Antennas Propagat..

[14] Yee A., Garbacz R. (1973). Self-and Mutual-Admittances of Wire Antennas in Terms of Characteristic Modes. IEEE Trans. Antennas Propagat..

[15] Kim G., Kim S. (2021). Design and Analysis of Dual Polarized Broadband Microstrip Patch Antenna for 5G mmWave Antenna Module on FR4 Substrate. IEEE Access.

[16] Adams J.J., Genovesi S., Yang B., Antonino-Daviu E. (2022). Antenna Element Design Using Characteristic Mode Analysis: Insights and Research Directions. IEEE Antennas Propag. Mag..

[17] Gao G., Zhang R.-F., Geng W.-F., Meng H.-J., Hu B. (2020). Characteristic Mode Analysis of a Nonuniform Metasurface Antenna for Wearable Applications. Antennas Wirel. Propag. Lett..

[18] Li H., Tan Y., Lau B.K., Ying Z., He S. (2012). Characteristic Mode Based Tradeoff Analysis of Antenna-Chassis Interactions for Multiple Antenna Terminals. IEEE Trans. Antennas Propagat..

[19] Mohanty A., Behera B.R. (2021). Characteristics Mode Analysis: A Review of Its Concepts, Recent Trends, State-Of-The-Art Developments and Its Interpretation with A Fractal UWB MIMO Antenna. Prog. Electromagn. Res. B.

[20] Balanis C.A. (2016). Antenna Theory: Analysis and Design.

[21] Ukkonen L., Sydanheimo L., Kivikoski M. (2005). Effects of Metallic Plate Size on the Performance of Microstrip Patch-Type Tag Antennas for Passive RFID. Antennas Wirel. Propag. Lett..

[22] Klionovski K., Shamim A. (2017). Back Radiation Suppression Through a Semitransparent Ground Plane for a Millimeter-Wave Patch Antenna. IEEE Trans. Antennas Propagat..

[23] Redmayne M., Maisch D.R. (2023). ICNIRP Guidelines’ Exposure Assessment Method for 5G Millimetre Wave Radiation May Trigger Adverse Effects. Int. J. Environ. Res. Public Health.

[24] Taguchi K., Kodera S., Hirata A., Kashiwa T. (2022). Computation of Absorbed Power Densities in High-Resolution Head Models by Considering Skin Thickness in Quasi-Millimeter and Millimeter Wave Bands. IEEE J. Electromagn. RF Microw. Med. Biol..

[25] IT’IS Foundation TISSUE DB. Database at a Glance. https://itis.swiss/virtual-population/tissue-properties/database/.

[26] Bommisetty L., Pawar S., Venkatesh T.G. (2022). Performance Analysis of Random Access Mechanism in 5G Millimeter Wave Networks: Effect of Blockage, Shadowing and Mobility. IEEE Access.

[27] Shariff B.G.P., Naik A.A., Ali T., Mane P.R., David R.M., Pathan S., Anguera J. (2023). High-Isolation Wide-Band Four-Element MIMO Antenna Covering Ka-Band for 5G Wireless Applications. IEEE Access.

[28] Iqbal A., Al-Hasan M., Mabrouk I.B., Nedil M. (2022). A Compact Implantable MIMO Antenna for High-Data-Rate Biotelemetry Applications. IEEE Trans. Antennas Propagat..

[29] Munir M.E., Al Harbi A.G., Kiani S.H., Marey M., Parchin N.O., Khan J., Mostafa H., Iqbal J., Khan M.A., See C.H. (2022). A New Mm-Wave Antenna Array with Wideband Characteristics for Next Generation Communication Systems. Electronics.

[30] Mneesy T.S., Hamad R.K., Zaki A.I., Ali W.A.E. (2020). A Novel High Gain Monopole Antenna Array for 60 GHz Millimeter-Wave Communications. Appl. Sci..

[31] Ali Esmail B., Koziel S. (2023). High Isolation Metamaterial-Based Dual-Band MIMO Antenna for 5G Millimeter-Wave Applications. AEU Int. J. Electron. Commun..

[32] Khan J., Ullah S., Ali U., Tahir F.A., Peter I., Matekovits L. (2022). Design of a Millimeter-Wave MIMO Antenna Array for 5G Communication Terminals. Sensors.

[33] Munir M.E., Kiani S.H., Savci H.S., Marey M., Khan J., Mostafa H., Parchin N.O. (2023). A Four Element Mm-Wave MIMO Antenna System with Wide-Band and High Isolation Characteristics for 5G Applications. Micromachines.

